# Stem Cell Therapy and Models for Autism Spectrum Disorder: Insights and Research

**DOI:** 10.2174/011570159X368403250618054913

**Published:** 2025-07-03

**Authors:** Yulong Liu, Yi Luo, Jiayin Liu, Meifeng Gong, Meiling Xia, Xiaotang Fan

**Affiliations:** 1 Department of Military Cognitive Psychology, School of Psychology, Third Military Medical University (Army Medical University), Chongqing, China

**Keywords:** Autism spectrum disorder, stem cells, cellular therapy, core symptom, stem cell transplantation, cell-based therapies

## Abstract

Autism Spectrum Disorders (ASD) are complex neurodevelopmental conditions characterized by impaired social communication, repetitive behavior patterns, and atypical sensory perception. The Autism and Developmental Disabilities Monitoring Network reports that approximately 1 in 36 children are diagnosed with ASD, highlighting the increasing prevalence and the pressing need for innovative treatment approaches. Medications commonly used in ASD primarily aim to manage associated symptoms, as there are currently no FDA-approved medications specifically for treating ASD core symptoms. Stem cells have demonstrated significant potential in cell-based therapies for ASD and have been utilized in *in vitro* models to investigate the pathogenesis of the condition. This review focuses on the recent advancements in stem cell-based transplantation in animal models of ASD, aiming to explore the improvement of ASD symptoms and the underlying mechanisms involved. It also discussed the application of stem cell-based transplantation in pediatric and adolescent populations with ASD to evaluate treatment efficacy and potential preventive strategies. Furthermore, recent efforts are addressed in developing stem cell-based models for both syndromic and non-syndromic forms of ASD, emphasizing studies that utilize cerebral organoids for modeling ASD, which facilitate the exploration of disease mechanisms within a tissue-like environment.

## 1 INTRODUCTION

Autism spectrum disorders (ASD) are neurodevelopmental disorders characterized by deficits in social interaction and communication, as well as stereotyped behaviors. It is crucial to recognize that each individual with ASD exhibits a unique combination of traits and symptoms, making their manifestations distinct [1, 2]. ASD typically appears in early childhood, with symptoms often becoming apparent before the age of 3 [3]. However, diagnosis may occur later, particularly in individuals with milder symptoms. The Centers for Disease Control and Prevention’s (CDC) Autism and Developmental Disabilities Monitoring (ADMM) Network estimates that around 1 in 36 children are diagnosed with ASD [4]. Given the considerable number of children with autism, it is crucial to provide early intervention services, therapies, and support systems to help individuals with ASD reach their full potential and improve their lives [5]. The management of ASD typically involves a multidisciplinary approach that focuses on behavioral and educational interventions, as well as addressing associated medical or mental health conditions, such as hyperactivity, aggression, or anxiety. However, at present, the FDA has not sanctioned any specific drug treatment for the primary symptoms of ASD [6]. There is an urgent requirement for new and better-targeted therapies.

The precise causes and development of ASD remain elusive, as a multifaceted mix of genetic and environmental elements contribute to its development risk [7-10]. It is estimated that hundreds of genes are involved in the development of autism, with specific genetic variations or mutations occurring more frequently in individuals with ASD compared to the general population. The heritability of autism can be demonstrated by some family and twin studies [11]. These studies consistently demonstrate that individuals with a family history of ASD are at a higher risk of developing the disorder themselves compared to the general population. The risk of ASD increases with increasing genetic relatedness to an affected individual. In addition to genetic factors, several environmental factors have been related to an increased risk of ASD, such as maternal infections during pregnancy [12], exposure to certain chemicals, and complications during pregnancy or birth [13]. It is important to recognize that the relationship between environmental factors and ASD is complex and not entirely elucidated. Notably, not all individuals exposed to these factors will develop ASD, indicating the multiple interacting elements contribute to the overall risk.

The mechanisms of ASD pathogenesis may involve changes in neuro-immunity, inhibited neurogenesis, imbalances in neurotransmission, maternal immune influences, *etc*. Notably, individuals with ASD often exhibit elevated levels of inflammatory cytokines such as TNF-α, IL-1β, and IL-6, which have been linked to behavioral abnormalities. Stem cells have shown promise in cell-based therapy for ASD due to their nutritional and immunomodulatory properties. Both preclinical and clinical studies have suggested the potential therapeutic benefits of stem cells for conditions like spinal cord injury, amyotrophic lateral sclerosis (ALS), and multiple sclerosis (MS) [14-17]. Specifically, stem cell-based therapy has been proposed as a promising approach for alleviating ASD [18]. Research has indicated that cell transplantation can support neurodevelopmental processes disrupted in individuals with ASD, such as neurogenesis, synaptogenesis, and neural network connectivity [19]. Additionally, a growing body of evidence suggests that stem cells offer a promising *in vitro* model for studying ASD pathogenesis [20]. This review aims to highlight the potential neurological benefits of stem cell therapy and explore the pathological and biological mechanisms underlying ASD in stem cell models.

## 2 METHODOLOGY

This study searched on PubMed, covering the period from the establishment of the database to August 2024. The keywords were combinations of various stem cells, including hematopoietic stem cell, neural progenitor cell, mesenchymal stem cell, bone marrow-derived mononuclear cell, fetal stem cell, umbilical cord blood cell, and autism or ASD, along with manual screening of reference lists from relevant review articles. The inclusion criteria were animal experiments or clinical studies that clearly utilized cell transplantation therapy as a treatment method. The exclusion criteria were articles with a publication type of conference abstract, review, editorial, incomplete data, or inaccessible full text. The retrieved articles were independently evaluated by two researchers to determine their eligibility based on the inclusion criteria. Any discrepancies were resolved through discussion or third-party adjudication. A standardized form was designed to extract the following variables: author, year, study type (animal experiment or clinical study), cell type, administration method, and treatment effect. Finally, the data was summarized into two primary tables.

## 3 STEM CELL TYPES FOR CELL THERAPY IN ASD

**Fig. (1):**
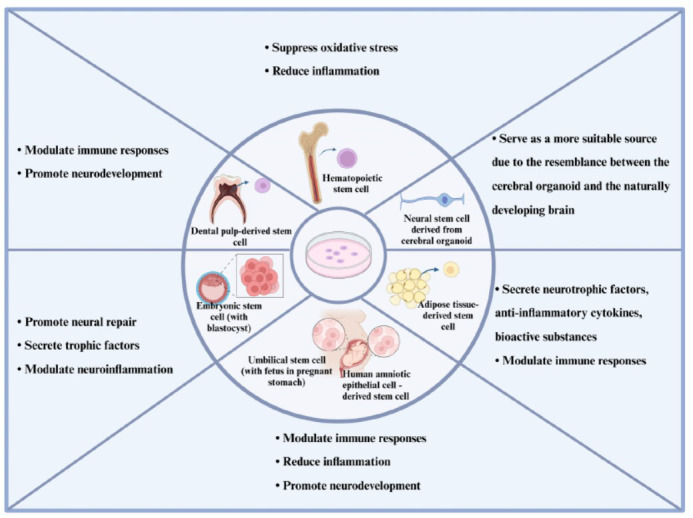
The cell types and mechanisms of ASD following treatment with different stem cells. The schematic includes representations of seven major candidate cell types: dental pulp-derived stem cell, hematopoietic stem cell, neural stem cell, embryonic stem cell, umbilical stem cell, human amniotic epithelial cell-derived stem cell, and adipose tissue-derived stem cell. These transplanted cells provide therapeutic benefits through various mechanisms, such as promoting neurodevelopment, secreting neurotrophic factors, modulating the immune system, and reducing inflammation.

Stem cells can be sourced from various origins, such as fetal or embryonic tissue, bone marrow, umbilical cord blood, adipose tissue (fat), and dental pulp [21]. Each type of stem cell possesses distinct characteristics and offers specific potential applications. Multiple categories of stem cells hold potential for therapeutic interventions in ASD (Fig. **1**).

### 3.1 Fetal or Embryonic Stem Cells

Stem cells in fetuses or embryos originate from the inner cell mass of embryos during the early stages of development. They can potentially develop into nearly all types of cells in the human body, making them valuable for regenerative medicine. Studies using embryonic stem cell (ESC) transplantation in rodent models of ASD have shown promising results [22]. The transplanted ESCs can differentiate into various cell types within the brain, potentially replacing damaged or dysfunctional cells and promoting neural repair [23].

### 3.2 Bone Marrow-derived Stem Cells

Bone marrow is a widely recognized source of adult stem cells, such as hematopoietic stem cells (HSCs) and mesenchymal stem cells (MSCs). HSCs play a crucial role in generating various blood cells and have been utilized in bone marrow transplants for decades to treat specific types of cancer and blood disorders [24]. Alongside HSCs, MSCs are also found in the bone marrow and can differentiate into fat, cartilage, and bone cells [25]. Ongoing research has explored the potential use of bone marrow-derived stem cells (BMSCs) in treating various conditions, including cardiovascular diseases [26], neurological disorders [27], and tissue injuries [28].

### 3.3 Umbilical Cord Blood-derived Stem Cells

Umbilical cord blood is an additional source of stem cells containing HSCs similar to bone marrow. It has been utilized in transplantation therapies for specific blood disorders such as leukemia [29], lymphoma [30], and certain genetic disorders impacting the blood and immune system [31]. Additionally, umbilical cord tissue contains MSCs with regenerative potential similar to bone marrow-derived MSCs [32]. The rationale behind umbilical cord blood-derived stem cells for ASD is that these stem cells may modulate immune responses, reduce inflammation, and promote neurodevelopment by secreting various factors that aid in tissue repair and neurogenesis [33].

### 3.4 Adipose Tissue-derived Stem Cells

Human adipose tissue-derived stem cells (hASCs) are a type of adult stem cells that can be found in fat tissue [34]. These cells are highly advantageous for therapeutic use due to their abundance in adipose tissue, can be easily isolated through minimally invasive procedures, and have the capacity to differentiate into various cell types, including neural cells [35]. hASCs can secret neurotrophic factors, anti-inflammatory cytokines, and other bioactive substances that support neuronal survival, promote neurogenesis, and modulate immune responses [36]. In the context of ASD, studies have explored the potential of hASCs to promote neurodevelopment and alleviate symptoms associated with the disorder [37].

### 3.5 Dental Pulp-derived Stem Cell

Dental pulp-derived stem cells (DPSCs) are a type of adult stem cell obtained from the soft tissue inside teeth with characteristics, such as the ability to differentiate into various cell types and possess immunomodulatory and regenerative properties [38]. DPSCs can be easily acquired and have shown promise in treating neurodegenerative disorders such as Alzheimer’s disease and Parkinson’s disease (PD) by being able to differentiate into neural cells and support damaged neurons [39]. In the field of ASD, research has explored the potential of DPSCs in modulating immune responses, promoting neurodevelopment, and alleviating symptoms associated with the disorder [40].

### 3.6 Human Amniotic Epithelial Cells-derived Stem Cells

Human amniotic epithelial cells (hAECs) are derived from the amniotic membrane surrounding the fetus during pregnancy. They offer numerous advantages for therapeutic use, including their non-controversial and abundant source immunomodulatory effects and potential to differentiate into various cell types. Additionally, they secrete factors that aid in tissue repair and possess anti-inflammatory properties [41]. A study has explored the potential of hAECs to regulate immune responses, promote neurodevelopment, and ameliorate ASD-related symptoms [42].

### 3.7 Cerebral Organoid-derived Stem Cells

Over several decades, stem cell research has had significant technological strides, particularly in generating cerebral organoids from induced pluripotent stem cells (iPSCs) and ESCs [43, 44]. Unlike conventional two-dimensional (2D) cultures, cerebral organoids undergo complex spatial and temporal regulation, making them more compatible with host cells for potential cell transplant treatments [45]. Stem cells derived from cerebral organoids can be extracted and grown in culture. These cells can self-renew and develop into different types of cells present in the organoid, such as neurons, astrocytes, and oligodendrocytes [46-48]. Given the resemblance between the cerebral organoid and the naturally developing brain [49], the brain precursor cells developed in organoid settings serve as a more suitable source for therapeutic purposes. Kim *et al.* have confirmed that transplantation of organoid-derived neural stem cells has yielded consistent and effective therapeutic results in animal models of PD. The culture of organoids *in vitro* also opens up the possibility of utilizing them as a source of stem cells for transplantation to treat ASD [50].

## 4 PRECLINICAL TRIALS WITH STEM CELLS IN ANIMAL MODELS OF ASD

**Fig. (2):**
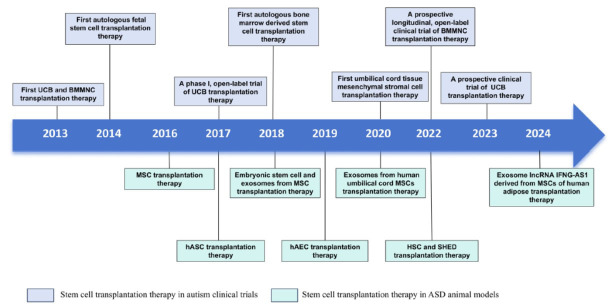
Timeline of landmark events related to stem cell transplantation in the therapy of ASD. This timeline outlines the advancements in stem cell transplantation therapy in clinical and preclinical trials for ASD. The abbreviations used include UCB for umbilical cord blood, BMMSC for bone marrow-derived mononuclear cells, MSC for mesenchymal stem cells, hASC for human adipose-derived stem cells, hAEC for human amniotic epithelial cells, HSC for hematopoietic stem cells, and SHED for stem cells from human exfoliated deciduous teeth.

The first transplantation therapy was introduced in 2016 using animal models of ASD. There are several ASD animal models, such as environment-induced models, idiopathic models, and genetic models, that have been generated based on human mutations and phenotypes that can be used in transplantation therapy. To assess the therapeutic effects, cultured stem cells are typically administered to the animals *via* intravenous or intraperitoneal injection. With further research, many have shown significant improvement in symptoms after receiving stem cell therapy. Meanwhile, more stem cells are being utilized in treatment (Table **1**, Fig. **2**).

### 4.1 Hematopoietic Stem Cell Transplantation in Maternal Diabetes-induced Autistic Mouse

It has been observed that the offspring from diabetic mouse mothers may exhibit behavioral abnormalities that resemble certain features of ASD, such as social interaction deficits, repetitive behaviors, and cognitive impairments [51, 52]. Zeng *et al*. found that the mouse offspring with autism triggered by maternal diabetes show specific epigenetic alterations in the superoxide dismutase 2 (SOD2) promoter, resulting in the inhibition of SOD2 in both HSC and peripheral blood mononuclear cells (PBMC) [53]. Further, it was confirmed that bone marrow transplantation of normal HSCs into offspring of mothers with diabetes-induced autism significantly reversed SOD2 suppression and oxidative stress in PBMC due to epigenetic inheritance from HSCs. Consistently, transplantation of SOD2-expressing HSCs partly reversed maternal diabetes–mediated SOD2 suppression in amygdala tissues as well as subsequent oxidative stress and autism-like behavior in the offspring.

### 4.2 Neural Progenitor Cell Transplantation in the Mouse Model of ASD

ASD individuals and animal models have a decrease in GABAergic interneurons [54], the levels of gamma-aminobutyric acid (GABA) [55, 56], and the enzymes glutamate decarboxylase (GAD) 65/67 [57, 58]. Donegan *et al*. conducted a study in which parvalbumin-positive interneurons were transplanted into the medial prefrontal cortex (mPFC) of a Poly I: C-induced autism rodent model. The results showed reduced firing activity in pyramidal cells and improved social interaction and cognitive flexibility, suggesting that restoring PV interneuron function in the mPFC could be a promising therapy for core ASD symptoms [22]. TRIM32 is a genetically associated risk gene for ASD [59]. Another study found that L/MGE progenitor transplantation rescues hyperexcitability and social deficits of TRIM32^-/-^ mice by enhancing GABAergic inhibition [60].

### 4.3 Mesenchymal Stem Cell Transplantation in ASD

MSCs possess regenerative and immunomodulatory properties that can be useful in treating ASD [61]. These secrete factors promote tissue repair, modulating inflammation and exerting neuroprotective effects. In a study using rats with autism induced by valproic acid (VPA), the intranasal administration of BM-MSCs conditioned medium (BM-MSCs-CM) significantly reduced stereotyped and repetitive behavior, inhibited microgliosis, decreased pro-inflammatory cytokines IL-1β and IL-6, and increased anti-inflammatory cytokine IL-10. The BM-MSC group showed a greater improvement than the BM-MSCs-CM group, highlighting the role of autocrine mechanisms in the therapeutic effects [62]. Meanwhile, Nikolai Gobshtis concludes that administering MSCs after development can help overcome prenatal neurodevelopmental deficits and restore cognitive and social behaviors by regulating hippocampal adult neurogenesis [63]. Hadar Segal-Gavish *et al.* revealed that a single intracerebroventricular human MSC transplantation in BTBR T+Itpr3tf/J (BTBR) mice exerted significant therapeutic effects involved in the elevated BDNF levels in the hippocampus and increased hippocampus neurogenesis [64]. Consistently, one study conducted by Jingyi *et al*. found that intravenous treatment with hBMMSC demonstrated positive effects on ASD [65]. Additionally, Perets *et al.* transplanted MSCs or NurOwn^®^ (MSCs with higher levels of BDNF and GDNF) into BTBR mice and found that single transplantation had a long-lasting positive effect on communication abilities, with NurOwn^®^ exhibiting superior benefits due to the induced neurotrophic factors [66]. Ha *et al.* confirmed the effectiveness of hASCs intraventricularly injected into neonatal mouse pups with VPA-induced autism in alleviating repetitive behaviors and social deficits [37]. The study further discovered that hASCs transplantation restored the altered expression of PTEN and the p-AKT/AKT ratio and rescued decreased levels of vascular endothelial growth factor and IL-10 in the brains of VPA-induced ASD model mice. This study demonstrated that hAEC transplantation alleviates social deficits in adult BTBR mice, restoring neurogenesis and neural progenitor cells (NPCs) in the hippocampus [42]. Additionally, several studies have demonstrated that the binding of BDNF to its receptor, TrkB, regulates hippocampal neurogenesis [67-69]. It was noted that BDNF and TrkB levels were restored in the hippocampus of hAECs-injected BTBR mice, likely contributing to improved neurogenesis. Zhao *et al.* found that administering SHED significantly improved the impaired social novel preference and social stress experienced by SHANK3 mutant beagle dogs *via* normalized the altered levels of interferon-γ and interleukin-10 in the serum [40].

### 4.4 Exosomes Secreted from MSC Transplantation in ASD

Exosomes, originating from MSCs, are organelles that contain bioactive molecules essential to the therapeutic effects observed in ASD treatment. Furthermore, MSCs can provide enduring effects through exosome secretion following transplantation. Compared to MSCs, exosomes secreted from MSC (MSC-exos) exhibit superior stability during storage and transportation. At the same time, their non-cellular nature confers critical advantages, including reduced immunogenicity, the elimination of oncogenic risks, enhanced clinical safety profiles, and simplified ethical considerations, making them more viable candidates for clinical translation. Perets *et al.* reported that intranasal administration of MSC-exo had significant beneficial effects on BTBR mice, improving their social and communicational phenotypes, increasing male-to-female ultrasonic vocalizations, and significantly enhancing maternal behaviors related to pup retrieval [70]. They also discovered that MSC-exo treatment had a direct positive impact on the autistic-like phenotype of the SHANK3 KO mice, enhancing social behavior in multiple paradigms, increasing vocalization, and reducing repetitive behaviors *via* the GABA-mediated pathway in the prefrontal cortex [71]. A recent study revealed that exosomes from hASCs can enhance neurogenesis in brain organoids and reduce social deficits in BTBR mice [72]. The lncRNA IFNG-AS1 in hASC-exos influences the miR-21a-3p/PI3K/AKT pathway, promoting neurogenesis [72]. Liang *et al.* demonstrated that intranasal delivery of exosomes derived from human umbilical cord MSCs restored social abilities, corrected repetitive and stereotypical behaviors, and alleviated various abnormal phenotypes in mouse models of ASD induced by VPA treatment *via* anti-inflammatory effects [73]. MSC-derived extracellular vesicles are similar to MSC-exo, and a study shows that they can ameliorate autism-like behaviors and inhibit pro-inflammatory factors in BTBR mice [74].

As mentioned above, a therapeutic strategy with transplanted stem cells has been demonstrated to be effective in alleviating ASD-like behaviors in ASD animal models. The contribution of preclinical studies has been highly successful in understanding the therapeutic effectiveness mechanism and carving the path toward clinical application.

## 5 CLINICAL TRIALS WITH STEM CELLS IN ASD PATIENTS

Clinically, the first transplantation therapy for ASD was reported in 2013. Since then, many studies have utilized stem cells for ASD treatment, demonstrating positive therapeutic outcomes (Table **2**, Fig. **2**).

### 5.1 Transplantation of Bone Marrow-derived Mononuclear Cells

Bone marrow-derived mononuclear cells (BMMNCs) encompass a diverse group, including HSCs, MSCs, endothelial progenitor cells (EPCs), small embryonic-like cells, and immune cells [75]. The potential therapeutic effects of BMMNC transplantation have been investigated for a range of conditions [76], such as stroke [77], traumatic brain injury [78], ALS [79], and spinal cord injury [80]. In a pioneering clinical study, Sharma *et al.* administered autologous BMMNCs through intrathecal transplantation to 32 ASD patients, followed by occupational therapy, speech therapy, and psychological intervention [81]. The results showed that 29 patients (91%) demonstrated improvement based on their total Indian scale for assessment of autism (ISAA) scores, 20 patients (62%) showed reduced severity on the Clinical Global Impression-Improvement (CGI-I) scale; on the CGI-II assessment, 96% of patients exhibited global improvement [81]. In a compelling case study, it was found that a 14-year-old boy with severe autism showed significant positive effects that persisted throughout the 1-year follow-up following the intrathecal transplantation of autologous BMMNCs into the L4-L5 spinal area [82]. Similar case reports indicate that combining BMMNC infusion with neurorehabilitation can lead to clinical improvements in ASD individuals, as evidenced by increases in ISAA, CGI, and Childhood Autism Rating Scale scores [83-88]. A clinical trial conducted at Vinmec International Hospital in Hanoi, Vietnam, from July 2017 to August 2019 showed that BMMNC transplantation could potentially improve the adaptive capacity of autistic patients who effectively tolerated the combination of BMMNC transplantation and behavioral intervention [89]. One non-randomized study found significant improvements in eye contact, attention, hyperactivity, social interaction, and communication among ASD patients, in which younger patients showed the most notable gains, highlighting the benefits of early intervention through autologous BMMNC and neurorehabilitation [90]. A recent study involving 24 participants found that intrathecal administration of BMMNC led to improved clinical outcomes by day 30, with these benefits continuing to enhance and sustained by day 180 post-treatment [91]. Crucially, it revealed a positive correlation between the clinical advantages of intrathecal BMMNC treatment and the increased presence of specific CD133^+^ stem cells.

### 5.2 Transplantation using Autologous Bone Marrow-derived Stem Cells

In a single randomized controlled trial involving 32 ASD children aged 5-15 years, they were randomly assigned to receive either autologous bone marrow MSCs (BMMSCs) combined with rehabilitation treatment and risperidone or only rehabilitation therapy and risperidone [92]. Although the intrathecal injection of autologous BMMSCs was found to be safe and feasible, its efficacy in treating ASD in children remains limited. Zakerinia *et al*. have demonstrated clinical improvements in some patients with PD, cerebral palsy, hypoxic brain damage, multiple sclerosis, and cerebellar atrophy following intrathecal autologous bone marrow-derived HSC therapy over the past six years in a single center; however, there were no observed improvements in patients with autism [93]. A study conducted by Kobinia *et al.* presented four cases of children with ASD aged 4 to 14 years who received intrathecal autologous bone marrow-derived stem cell transplants [94]. The procedure was well-tolerated without any significant adverse effects in the short or long term. There was a noticeable improvement in symptoms of ASD, reflected in better scores on the Autism Treatment Evaluation Checklist (ATEC) in the year after the transplant [94]. In some cases of ASD, the immune system and inflammation are activated abnormally within the brain [95-97]. One possible mechanism for the positive effect of the transplant is its potential to counteract a cerebral inflammatory autoimmune process.

### 5.3 Transplantation using Fetal Stem Cells

An open-label pilot study by Bradstreet *et al.* with 39 males and 6 females aged 3-15 examined fetal stem cell treatment for ASD [98]. Significant improvements were noticed in 78% of participants, such as calmness, eye contact, and appetite, along with enhanced ATEC/ABC scores for speech and sociability, sensory, and overall health. Importantly, no side effects were observed in any of the subjects who received the fetal stem cells, and there were no cases of transmittable diseases for up to a year after treatment. It is hypothesized that paracrine and immunomodulatory effects of fetal stem cells can help restore immune balance, dampen excessive inflammation, and promote neurodevelopment.

### 5.4 Transplantation using Umbilical Cord Blood Cells

A phase I/II trial at a single center evaluated the effectiveness and safety of transplanting human cord blood mononuclear cells (CBMNCs) and umbilical cord-derived mesenchymal stem cells (UCMSCs) in 37 children with ASD [99]. This non-randomized, open-label study involved weekly administration of CBMNCs alone or with UCMSCs for four sessions, of which the combined treatment significantly enhanced core ASD symptoms and overall clinical outcomes [99]. Dawson and colleagues conducted a preliminary study involving 25 young autistic children (aged 2-6), evaluating the safety and feasibility of an intravenous infusion of autologous umbilical cord blood for phase I clinical trials [33]. The results showed no adverse events over 12 months and improved behavior, particularly in children with higher baseline nonverbal IQs. Notably, there were significant changes in EEG spectral characteristics, including increased alpha and beta power and decreased theta power, with higher posterior beta power linked to improvements in social communication symptoms, suggesting that EEG biomarkers predict outcome variability. The disparity is in a subsequent study, including 180 children with ASD (aged 2-7) receiving a single intravenous autologous or allogeneic umbilical cord blood infusion, which showed that the treatment did not improve socialization skills or reduce autism symptoms [100]. However, a sub-analysis of children without intellectual disability showed positive effects on communication skills, exploratory measures, and increased α and β EEG power [101]. In a clinical trial examining the effects of autologous umbilical cord blood (AUCB) infusion on children with ASD, 29 participants aged between 2 and 6 years were randomly selected and divided into a blinded, placebo-controlled group [102]. There were some promising improvements in socialization skills, with no notable disparities between the groups, suggesting that AUCB infusions are a safe option for treating ASD [102]. Petriv *et al.* found that administering umbilical cord stem cells through intrathecal and intravenous methods in children with ASD resulted in positive changes in behavior within two weeks of the procedure without significant adverse reactions [103]. AUCB may help reduce neuroinflammation and develop white matter in the brain, leading to changes in connectivity patterns [104, 105]. Intravenous administration of AUCB has been found to enhance social and communication skills and increase white matter connectivity in brain regions responsible for these functions [106]. Ricci curvature was also used to measure changes in brain networks following AUCB infusion, revealing consistency in identifying regions of the brain correlated with improvements in social communication [107]. A recent study revealed that the CARS score fell below the threshold four years after AUCB therapy in autistic patients. Analysis of the patients' blood samples indicated a downregulation of pro-inflammatory gene expressions, particularly TNF-α and IL-1β. This suggests the potential of AUCB therapy in mitigating neuroinflammation and enhancing symptoms of ASD [108].

### 5.5 Transplantation using Umbilical Cord Tissue Mesenchymal Stromal Cells

In a phase I study, 12 children aged 4 to 9, diagnosed with ASD, received treatment through the intravenous infusion of human cord tissue mesenchymal stromal cells (hCT-MSCs) [109]. The children received one, two, or three doses of the hCT-MSCs at 2-month intervals. The children were evaluated clinically and through laboratory tests at the beginning of the treatment and six months later in person, with a remote evaluation conducted 12 months after the final infusion. Overall, the treatment was generally well-tolerated, and 6 of the participants showed improvement in at least two measures specific to ASD, indicating a positive response to the treatment in a subset of participants [109].

Taken together, stem-cell therapy is a promising treatment option for patients diagnosed with ASD. The mode of transmission, stem cell type, and dose of stem cells is imperative in result outcomes. This therapy seemed to be safe for different age groups, but the long-term safety and efficacy of stem cell therapy are still being adequately investigated.

## 6 STEM CELL-DERIVED PLATFORMS FOR ASD MODELING

### 6.1 Two-dimensional Culture System

Alterations in gene expression patterns during neurodevelopment may contribute to the development and manifestation of ASD symptoms. The transformation of healthy human neuronal progenitors into mature, non-dividing neurons can lead to gene expression patterns associated with ASD. Recently, hESCs and hiPSCs, derived from somatic cell reprogramming, have been valuable for modeling ASD-related conditions [110].

Rett syndrome is a neurodevelopmental condition primarily caused by mutations in the MECP2 gene, featuring symptoms similar to ASD, such as cognitive dysfunction, movement impairments, and social interaction deficits [111]. It has been confirmed that neurons derived from Rett syndrome-iPSCs exhibit reduced synapses, smaller soma size, altered calcium signaling, and electrophysiological defects, which recapitulate the early stages of ASD [111]. Mutant Rett syndrome-hiPSC-derived neurons also exhibit similar phenotypes, supporting findings from neuroimaging and postmortem studies in individuals with ASD. Additionally, the expansion of the CGG trinucleotide repeat in the FMR1 gene causes fragile X syndrome (FXS), a genetic disorder resulting in the loss of FMR protein (FMRP), intellectual disability, and ASD [112]. The abnormal synaptic activity observed in FXS iPSC-derived neurons aligns with the characteristic synaptic dysfunction of FXS. Specifically, the absence of FMRP delays the developmental switch from GABA’s excitatory to inhibitory function, which may contribute to the excitatory-inhibitory imbalance observed in FXS [112].

Individuals with SHANK3 microdeletions have shown synaptic and morphogenetic anomalies in neurons derived from iPSCs. To gain deeper insights into the cellular and molecular mechanisms associated with ASD, Deneault *et al.* generated 53 iPSC-derived glutamatergic neuronal lines from 25 participants across 12 unrelated families with ASD [113]. Their findings revealed that glutamatergic neurons deficient in one copy of CNTN5 or EHMT2 displayed heightened neuronal activity, potentially elucidating specific ASD-related phenotypes [113]. Furthermore, the tuberous sclerosis complex (TSC) is strongly associated with ASD. Martin *et al.* found that iPSC-derived NPCs using CRISPR/ Cas9-mediated TSC1 loss exhibited enlarged cell size [114]. Wang *et al.* found that NPCs derived from hiPSCs of ASD individuals with early developmental brain enlargement had a higher level of inherent proliferation and displayed a modified DNA replication program and increased DNA damage [115]. A study by Marchetto found that iPSC-derived NPCs from individuals with idiopathic autism exhibited increased cell proliferation due to dysregulation of a β-catenin/BRN2 transcriptional cascade, and iPSC-derived neurons displayed reduced synaptogenesis, leading to functional deficiencies in neuronal networks [116]. Consistent with this research, hiPSC-derived neurons from ASD individuals demonstrated decreased capacity for cortical differentiation and abnormal calcium signaling [117, 118]. In addition, co-culture experiments involving 2D iPSC-derived neurons and astrocytes have revealed that ASD-derived astrocytes can impede proper neuronal development, and control-derived astrocytes have been found to rescue the neuronal morphology and synaptogenesis defects in ASD neuronal co-cultures [119]. Nevertheless, it is essential to note that the 2D culture system has limitations, as it cannot fully replicate the *in vivo* processes involved in ASD pathologies.

### 6.2 Three-dimensional (3D) Cerebral Organoid Culture System

Cerebral organoids are advanced 3D models created *in vitro* that mimic the structural and functional properties of the human brain and can be used to explore brain development and disease [120]. It is difficult to study human embryonic brain development directly because of the scarcity and inaccessibility of tissue from many developmental stages in ASD patients, as well as ethical restrictions. The cerebral organoid model has emerged as a valuable tool for studying neurodevelopmental disorders, including ASD [120]. Cerebral organoids have been used to model ASD and related comorbidities and have the potential to address critical cellular and molecular processes that may contribute to ASD development [121].

Mariani *et al.* utilized cerebral organoids to examine neurodevelopmental differences in severe idiopathic ASD patients and found that FOXG1, linked to ASD development, influenced a shift towards GABAergic neuron fate [122]. Another study conducted by Jourdon *et al.* used cerebral organoids and single-cell transcriptomics to find that transcriptomic changes in individuals with ASD with macrocephaly or porencephaly may result in disruptions to the equilibrium between excitatory neurons in the dorsal cortical plate and other neuronal lineages, such as early-generated neurons from the putative prelate [123]. The CRISPR–human organoids–single-cell RNA sequencing (CHOOSE) system, created by Li *et al*., has revealed 36 ASD high-risk genes involved in transcriptional regulation, with the most affected cell types being dorsal intermediate progenitors, ventral progenitors, and upper-layer excitatory neurons [124]. Additionally, cerebral organoids created from individuals with ASD have exhibited notable differences in energy metabolism, with increased glycolysis compared to oxidative phosphorylation, as well as variations in cell adhesion proteins, cell cycle elements, cytoskeleton, and multiple transcription factors.

Wang *et al.* used cerebral organoids derived from iPSCs to analyze RNA-seq on CHD8^+/−^ and isogenic control (CHD8^+/+^). The findings revealed a noteworthy overlap between the differentially expressed genes of CHD8^+/−^ and those identified in a separate transcriptome analysis conducted by a different group, which used cerebral organoids derived from a family with idiopathic ASD [125]. Bruna Paulsen *et al.* utilized human organoid models to confirm cell type-specific abnormal development resulting from haploinsufficiency in three ASD risk genes: SUV420H1 (KMT5B), ARID1B, and CHD8, affecting the asynchronous development of typical neurons [121]. Villa *et al.* conducted a study that revealed how CHD8 haploinsufficiency disrupts the development of the nervous system, leading to an imbalance between the generation of inhibitory and excitatory neurons and resulting in an abnormal enlargement of cerebral organoids consistent with the macrocephaly demonstrated in patients [126]. Astorkia *et al.* found that genes enriched with neural and brain development functions, cilium organization, and extracellular matrix organization were detected in CHD8 knockout cerebral organoids using single-cell RNA-seq analysis, and interactions between neural and glial cells were also affected [127]. In a study by Fair *et al*., a cerebral organoid model was used to examine the pathophysiology of a heterozygous *de novo* missense autism susceptibility candidate 2 (AUTS2) variant in a patient with primary microcephaly and severe intellectual disability, among other neurological impairments [128]. According to reports, 16p11.2 microdeletions are genetically associated with 1% of ASD cases. Fetit *et al*. suggest that these microdeletions cause increased developmental variability in organoids, disrupting typical development [129]. Alterations in the cell cycle and premature differentiation of ventral progenitors into interneurons may affect neuronal circuit connectivity, contributing to ASD symptoms. Thus, 16p11.2 microdeletions could play a role in ASD etiology by interfering with development. Wang *et al.* have shown that a single organoid derived from neural rosettes with a hemizygous deletion of the SHANK3 gene exhibits intrinsic and excitatory synaptic deficits [130]. Patients with a homozygous protein-truncating mutation in CNTNAP2 display clinical characteristics of brain overgrowth. The CNTNAP2-organoid model displayed an increase in volume and total cell number driven by increased neural progenitor proliferation, which can be corrected through mutation correction using CRISPR-Cas9 [131]. A study using a human cerebral organoid model with a microglia-incorporated SCN2A protein-truncating mutation, common in children with ASD, found that microglia increased the elimination of post-synapses in these organoids [132].

Maternal immune system activation (MIA) during pregnancy may affect fetal brain development, raising the risk of ASD. In a study with dorsal forebrain organoids treated with Hyper-IL-6, radial glial cells increased while genes related to protein translation were downregulated [133]. VPA exposure can increase the risk of ASD. Transcriptome analysis showed that the genes altered in VPA-exposed organoids resembled those in postmortem ASD brains and organoids from individuals with autism [134]. Meng *et al.* identified changes in gene expression in human forebrain organoids exposed to VPA, particularly in pathways related to neural development, synaptic transmission, and signaling mechanisms associated with ASD [135]. Using a microelectrode array, the authors also confirmed that VPA exposure disrupted synaptic transmission in forebrain organoids [135]. The analysis of this study shows that VPA exposure significantly affects genes linked to ASD risk and reveals that the Wnt/β-catenin signaling pathway is involved in cortical neurogenesis and oRGs output [136]. These results highlight the value of using dorsal forebrain organoids to model the teratogenic pathways induced by VPA during cortical development, which may contribute to ASD development.

## 7 FUTURE PERSPECTIVES AND CHALLENGES

Rapid advancements in stem cell therapy have highlighted the potential for treating ASD. While these studies are still in their early stages, they present promising opportunities for future therapeutic options. It is important to emphasize that further research requires more rigorous clinical trials with larger sample sizes and extended follow-up periods to determine the safety, efficacy, optimal cell type, dosage, and treatment duration of stem cell therapies for ASD. Considering the highly heterogeneous nature of ASD, using models derived from hiPSCs as a predictive platform could significantly enhance precision medicine development. This “clinical trials in a dish” approach could lead to more effective therapeutics for ASD individuals. ASD onset is linked to abnormalities occurring during early embryonic brain development. Cerebral organoids created from hESCs and hiPSCs show great promise for modeling ASD-related conditions and addressing challenges related to the scarcity and inaccessibility of tissue from various developmental stages in ASD patients, as well as ethical concerns. The cerebral organoid model highlights the critical need for advanced *in vitro* systems that accurately simulate human brain development and maturation [137]. These organoids modeling ASD and related comorbidities have the potential to elucidate essential cellular and molecular processes that may contribute to the onset of ASD [127]. Researchers continuously refine these organoids through genetic engineering techniques to improve cell therapies' safety, efficacy, and customization for individual patients.

## CONCLUSION

In conclusion, stem cell therapy shows potential as a viable treatment for ASD. Further clinical research is essential, necessitating more rigorous trials to assess the clinical benefits and safety for individuals with ASD. Additionally, pre-clinical studies and *in vitro* research are crucial for gaining a comprehensive understanding of the mechanisms of the therapeutic effects of stem cell transplantation.

## STUDY LIMITATIONS

Several limitations should be acknowledged. First, our search was restricted to PubMed, potentially missing relevantstudies in other databases. Second, stem cell therapy for ASD is a rapidly evolving field. However, literature publishedafter August 2024 could not be included. Additionally, while the organoid field encompasses extensive research on ASD, our article only discusses a subset of classical studies, which may not provide a comprehensive perspective on the topic.

## Figures and Tables

**Table 1:** Preclinical trials related to cellular therapy in animal models of ASD.

**Animal**	**Source**	**Results**	**References**
BTBR male mice aged 6-7 weeks	Bone marrow- derived MSC	Improves social deficits, reduces stereotypical behaviors, modifies microbiota compositions.	[65]
BTBR 4-week-old mice	Exosome lncRNA IFNG - AS1 derived from MSC of human adipose	Ameliorates neurogenesis and ASD-like behavior in BTBR mice.	[72]
The male pups of VPA-treated mice	BM-MSCs and BM-MSCs-CM	Alleviate prenatal mental deficits, restore cognitive and social deficits, and modulate microglial and inflammatory markers.	[62]
ddSHANK3 mutant beagle dogs, 5-7 months	Stem cells from human exfoliated deciduous teeth	Alleviate autistic-like symptoms of impaired SNP and obvious social stress in SHANK3 mutant.	[40]
Maternal diabetes mouse 6 weeks, male	HSC with increased SOD2 expression	Ameliorate maternal diabetes-mediated GI symptoms and autism-like behavior.	[53]
Male mice, 8-10 weeks; Shank3B KO line	Exosomes from MSC	Improve core symptoms of genetically modified mouse model of autism Shank3B.	[71]
The offspring of VPA-treated mice	Exosomes from human umbilical cord MSCs	Restore social ability and correct repeated stereotyped behaviors and other abnormal phenotypes.	[73]
TRIM32^−/−^ mice	L/MGE progenitor	Rescues hyperexcitability and social deficits.	[60]
BTBR male mice, 7-8 weeks	Human amniotic epithelial cells	Promotes neurogenesis and ameliorates social deficits.	[42]
BTBR mice, 4- week-old	Exosomes from MSC	Ameliorates autistic-like behaviors of BTBR mice.	[70]
BTBR mice, 4- week-old	MSC-derived extracellular vesicles	Ameliorate autism-like behaviors and inhibits pro-inflammatory factors.	[74]
Pregnant Sprague -Dawley rats (gestational day 11)	The J27 mouse embryonic stem cell line containing dual reporters	Normalize dysregulated cortical activity and reverse behavioral deficits relevant to autism.	[22]
BTBR 6-8- week-old mice	MSC secreting higher amount of neurotrophic factors (NurOwn^®^)	NurOwn^®^transplantation had long lasting effect of alleviating autistic behaviors in BTBR mice.	[66]
VPA-induced ASD mice, P2 or P3	Human adipose- derived stem cells	Ameliorates repetitive behavior, social deficit and anxiety.	[37]
VPA-treated mouse offspring	bone marrow-derived MSCs	Reverses behavioural deficits and impaired neurogenesis.	[63]
BTBR male mice (6-8 weeks of age)	MSC produced higher levels of neurotrophic factors	Reduces stereotypic behavior and cognitive rigidity; improves social deficits and communication skills.	[64]

**Abbreviations**: ASD, autism spectrum disorder; BTBR, BTBR T+ tf/J; MSCs, mesenchymal stem cells; lncRNA, long non-coding RNA; VPA, valproic acid; BM-MSCs, bone marrow-derived mononuclear cells; BM-MSCs-CM, BM-MSCs conditioned medium; SNP, social novelty preference; HSC, hematopoietic stem cells; SOD2, superoxide dismutase 2; GI, gastrointestinal; TRIM32, tripartite motif protein 32; M/LGE, medial/lateral ganglionic eminence.

**Table 2:** Clinical trials of cellular therapy in ASD patients.

**Study Design**	**Administration Route**	**Results**	**References**
**Transplantation Bone Marrow-derived Mononuclear Cells**			
An improved case of autism	Intrathecal	Symptomatic improvement with shift on CARS from 42.5 (Severely Autistic) to 23.5 (Non Autistic).	[82]
A case of adult autism	Intrathecal	At the 6th and 9^th^ month objective outcome measures of ISAA and CGI showed significant improvement.	[83]
An open label proof of concept study	Intrathecal	A total of 29 patients improved on total ISAA scores and 20 patients showed decreased severity on CGI-I. On CGI-II 96% patients showed global improvement.	[111]
A case of autism with comorbid mental retardation	Intrathecal	There was significant clinical improvement in social relationship, communication and behavior.	[84]
A 7-year-old boy with autism	Intrathecal	On regular follow ups conducted at the 3^rd^ and 6th month post-treatment, clinically significant behavioral, social, communication and cognitive improvements were reported.	[85]
A case of a six and a half-year-old boy with autism	Intrathecal	On follow up at 3^rd^ months and 7^th^ months post intervention, the patient showed significant symptomatic improvements with no major side effects	[86]
A 25-year-old male diagnosed with ASD at the age of 7 years	Intrathecal	Following six months of cell therapy, the patient showed improvement in concentration, attention, command following, sitting tolerance, social interactions, eye contact, and memory.	[87]
An open label non-randomized study	Intrathecal	Improvements in eye contact, attention and concentration, hyperactivity, sitting tolerance, social interaction, stereotypical behavior, aggressiveness, communication, speech, command following and self-stimulatory behavior.	[112]
A prospective, longitudinal, open- label clinical trial	Intrathecal	The patients showed a statistically significant reduction (improvement) in CARS scores after the treatment	[88]
Controlled and noncontrolled, randomized and non-randomized trials	Intravenous and intrathecal	Social communication, language, and daily skills improved. Repetitive behaviors and hyperactivity decreased remarkably.	[89]
A prospective longitudinal, open-label clinical trial	Intrathecal	Improved clinical outcomes by Day 30 and Day180	[91]
**Transplantation using Autologous Bone Marrow Derived Stem Cells**			
A randomized controlled trial	Intravenous and subcutaneous administered	Significant regarding the CGI-severity of illness, showing more pronounced improvement in the intervention group	[92]
Case reports: Ages of 4-14 years (three boys and one girl)	Intrathecal and simultaneously intravenous	Disappearance of symptoms during the following year and consequently improved (ATEC) scores	[94]
A single center study	Intrathecal	There were no improvements in patients	[93]
**Transplantation using Autologous Fetal Stem Cells**			
An open-labeled pilot study	Intravenously and subcutaneously administered	Significant improvements in 78% of participants, significant differences in ATEC/ABC scores	[98]
**Transplantation using Umbilical Cord Blood Cells**			
A randomized, double- blinded, placebo -controlled trial	Intravenous	Improvement on the Socialization Subscale of the Vineland	[102]
A phase I, single-center, and open-label trial	Intravenous	Higher baseline posterior EEG beta power was associated with a greater degree of improvement in social communication symptoms	[101]
A prospective clinical trial	Intravenous	Alleviation of autism with findings of anti-inflammatory response in his peripheral blood after UCB infusion.	[108]
Non-randomized, open-label, single center phase I/II trial	Intravenous and intrathecal	More prominent impact on core ASD symptoms and overall clinical presentation.	[32]
A phase I, open-label trial	Intravenous infusion	Behavioral improvements were observed during the first 6 months	[33]
A prospective, randomized, placebo- controlled, double-blind study	Intravenous	A sub-analysis of children without intellectual disability showed positive effects on communication skills, exploratory measures, and increased α and β electroencephalographic power.	[100]
An open label clinical trial	Intravenous	Changes in the curvature of the connections between regions associated with ASD	[107]
Clinical Investigation	Intrathecal	Resulted in positive behavioral changes within the first two weeks after the procedure	[103]
A phase I open-label trial	Intravenous	Improvements in social communication skills and a reduction in symptoms in children with ASD following treatment	[106]
**Transplantation using Umbilical Cord Tissue Mesenchymal Stromal Cells**			
An open-label, phase I study	Intravenous	Six of 12 participants demonstrated improvement in at least two ASD-specific measures.	[109]

**Abbreviations**: ASD, autism spectrum disorder; CARS, Childhood Autism Rating Scale; ISAA, Indian Scale for Assessment of Autism; CGI, Clinical Global Impression; ATEC, Autism Treatment Evaluation Checklist; ABC, Aberrant Behavior Checklist; EEG, electroencephalography; UCB, umbilical cord blood.

## LIST OF ABBREVIATIONS

ALS: Amyotrophic Lateral Sclerosis
ASD: Autism Spectrum Disorders
ATEC: Autism Treatment Evaluation Checklist
AUCB: Autologous Umbilical Cord Blood
BDNF: Brain-derived Neurotrophic Factor
BM-MSCs-CM: BM-MSCs Conditioned Medium
BMMNCs: Bone Marrow-derived Mononuclear Cells
BMMSCs: Bone Marrow Mesenchymal Stem Cells
BMSCs: Bone Marrow Derived Stem Cells
BTBR: BTBR T+Itpr3tf/J
CARS: Childhood Autism Rating Scale
CBMNCs: Cord Blood Mononuclear Cells
CGI-I: Clinical Global Impression-improvement
CHOOSE: CRISPR-human Organoids-single-cell RNA Sequencing
DPSCs: Dental Pulp-derived Stem Cells
EEG: Electroencephalography
EPCs: Endothelial Progenitor Cells
ESCs: Embryonic Stem Cells
FDA: Food and Drug Administration
FMRP: FMR Protein
GABA: Gamma-aminobutyric Acid
GAD: Glutamate Decarboxylase
GARS-II: Gilliam Autism Rating Scale-second Edition
hAECs: Human Amniotic Epithelial Cells
hASCs: Human Adipose-derived Stem Cells
hCT-MSCs: Human Cord Tissue Mesenchymal Stromal Cells
HSCs: Hematopoietic Stem Cells
hUC-MSCs: Human Umbilical Cord MSCs
iPSCs: Induced Pluripotent Stem Cells
ISAA: Indian Scale for Assessment of Autism
MIA: Maternal Immune System Activation
mPFC: Medial Prefrontal Cortex
MS: Multiple Sclerosis
MSC-exo: Exosomes Secreted from MSC
MSCs: Mesenchymal Stem Cells
NPCs: Neural Progenitor Cells
PBMC: Peripheral Blood Mononuclear Cells
PD: Parkinson’s Disease
PTEN: Phosphatase and Tensin Homolog
PV: Parvalbumin-positive
SHED: Stem Cells From Human Exfoliated Deciduous Teeth
SOD2: Superoxide Dismutase 2
TSC: Tuberous Sclerosis Complex
UCMSCs: Umbilical Cord-derived Mesenchymal Stem Cells
VPA: Valproic Acid
